# Identification of ankyrin-transmembrane-type subfamily genes in Triticeae species reveals *TaANKTM2A-5* regulates powdery mildew resistance in wheat

**DOI:** 10.3389/fpls.2022.943217

**Published:** 2022-07-22

**Authors:** Ping Hu, Yueming Ren, Jun Xu, Qichao Wei, Puwen Song, Yuanyuan Guan, Huanting Gao, Yang Zhang, Haiyan Hu, Chengwei Li

**Affiliations:** ^1^Henan Engineering Research Center of Crop Genome Editing, Henan International Joint Laboratory of Plant Genetic Improvement and Soil Remediation, College of Life Science and Technology, Henan Institute of Science and Technology, Xinxiang, China; ^2^College of Landscape Architecture and Horticulture, Henan Institute of Science and Technology, Xinxiang, China; ^3^College of Biological Engineering, Henan University of Technology, Zhengzhou, China

**Keywords:** ankyrin-transmembrane protein, evolutionary progress, powdery mildew, virus-induced gene silencing, wheat

## Abstract

The ankyrin-transmembrane (ANKTM) subfamily is the most abundant subgroup of the ANK superfamily, with critical roles in pathogen defense. However, the function of ANKTM proteins in wheat immunity remains largely unexplored. Here, a total of 381 *ANKTMs* were identified from five *Triticeae* species and *Arabidopsis*, constituting five classes. Among them, class a only contains proteins from Triticeae species and the number of ANKTM in class a of wheat is significantly larger than expected, even after consideration of the ploidy level. Tandem duplication analysis of *ANKTM* indicates that *Triticum urartu*, *Triticum dicoccoides* and wheat all had experienced tandem duplication events which in wheat-produced *ANKTM* genes all clustered in class a. The above suggests that not only did the genome polyploidization result in the increase of *ANKTM* gene number, but that tandem duplication is also a mechanism for the expansion of this subfamily. Micro-collinearity analysis of Triticeae *ANKTMs* indicates that some *ANKTM* type genes evolved into other types of *ANKs* in the evolution process. Public RNA-seq data showed that most of the genes in class d and class e are expressed, and some of them show differential responses to biotic stresses. Furthermore, qRT-PCR results showed that some *ANKTMs* in class d and class e responded to powdery mildew. Silencing of *TaANKTM2A-5* by barley stripe mosaic virus-induced gene silencing compromised powdery mildew resistance in common wheat Bainongaikang58. Findings in this study not only help to understand the evolutionary process of *ANKTM* genes, but also form the basis for exploring disease resistance genes in the *ANKTM* gene family.

## Introduction

Wheat (*Triticum aestivum* L.) is a leading source of calories for the global human diet (Schilling et al., 2020). Changes in the climate and crop planting systems have not only caused environmental degradation, but also the prevalence of diseases (Guo et al., 2010; Cui et al., 2018). In the winter wheat area, the proportion of powdery mildew in the total sown area has increased yearly with the change in climate (Tang et al., 2017). The most environmentally friendly way to control plant fungal diseases is to mine the gene pool for resistance and the cultivation of disease-resistant varieties (Kuraparthy et al., 2007).

The ankyrin repeat (ANK) is a 33-residue, two alpha helix motifs in proteins and is one of the most common protein domains present in bacteria, virus, plants and humans (Sedgwick and Smerdon, 1999; Mosavi et al., 2004). In ANK-containing proteins, the number, spatial structure and primary sequence of the ANK domains can differ substantially, and ANK proteins have been found to play diverse biological functions, usually involving ANK domain-mediated protein–protein interactions (Shen et al., 2010; Fu et al., 2012; Vo et al., 2015). However, ANK repeat domain-containing proteins can also contain other domains, including zinc or ring fingers, calmodulin binding or transmembrane domains, thus forming the structurally and functionally diverse ANK protein superfamily (Becerra et al., 2004). Structurally, the most abundant protein subgroup contains ankyrin-transmembrane domains (ANKTM; Becerra et al., 2004) and functionally, *ANKTM* family genes have been described with roles in plant development, hormone signal transduction, but especially in the attenuation of abiotic and biotic stress (Vo et al., 2015).

Previous studies of the ANKTM proteins showed that these can interact with different ligands to participate in important physiological and developmental processes. For example, TIP1 from *Arabidopsis*, which contains ANK, TM and DHHC domains, the latter of which confers S-acyl transferase activity involved in the positive regulation of root hair formation (Hemsley et al., 2005). ANK1 from *Nicotiana tabacum* interacted with BZI-1 and BZI-2 in the nucleus, and is involved in both auxin signaling and the pathogen response (Kuhlmann et al., 2003). ANKTM proteins are also related to plant-microorganism interactions and abiotic stress, the ANKTM protein, IGN1 of *Lotus japonicas*, is involved in symbiotic nitrogen fixation in root nodules and the *ign1* mutant plant grows abnormally due to nitrogen deficiency (Kumagai et al., 2007). *ITN1*, an *Arabidopsis* homolog of *IGN1*, is related to salt-stress tolerance through its effect on abscisic acid-induced production of reactive oxygen species under salt stress (Sakamoto et al., 2008).

In recent years, an increasing number of studies have shown that *ANKTMs* are involved in plant resistance to pathogens. The *Arabidopsis ANKTM* family gene *BDA1* is related to plant disease resistance, and acts downstream of the receptor-like protein *SNC2* and upstream of *NPR1* and *WRKY70* to regulate plant immunity (Yang et al., 2012). The ANKTM protein GBP, plays a role in the connection between defense response and carbohydrate metabolism and a loss-of-function mutant of *gbp* causes necrotic lesions (Wirdnam et al., 2004). *ACCELERATED CELL DEATH6* (*ACD6*) is a widely studied *ANKTM* gene that play an important role in broad-spectrum resistance in *Arabidopsis* (Lu et al., 2005; Todesco et al., 2010). *ACD6* acts in the plant immune response and is involved in salicylic acid signaling and salicylic acid-dependent cell death and defense (Rate et al., 1999; Lu et al., 2003). The *Arabidopsis* nucleotide-binding domain and leucine-rich-repeat-containing (NLR) resistance protein, *SNC1*, can modulate *ACD6*-dependent hyper immunity and link different arms of the plant immune system (Zhu et al., 2018). ACD6 can not only interact with pattern recognition receptors like BAK1 and CERK1 to form large complexes at the membrane (Tateda et al., 2014; Zhang et al., 2014b), but also regulates PAD4 and EDS1 to trigger the hypersensitive response (Feys et al., 2001). *ZmACD6*, the orthologous gene of *ACD6* in *Zea mays*, confers resistance to *Ustilago maydis* (Zhang et al., 2019). Recently, the race-specific leaf rust resistance gene, *Lr14a*, from hexaploid wheat was shown to encode an ANKTM protein (Kolodziej et al., 2021). A further study showed that the wheat stripe rust resistance protein, YrU1, is an NLR protein with an integrated ANK domain, and the ANK domain of YrU1 is derived from ANKTM proteins (Wang et al., 2020; Kolodziej et al., 2021). The above studies suggest that several *ANKTM*-type genes are involved in basal resistance or effector-triggered immunity.

The gene expansion, evolution, expression pattern and function of *ANKTM* genes in Triticeae species remain largely unexplored. In this study, based on whole genome information of wheat and its related species, a new subfamily of the ANKTM-type proteins in Triticeae species was identified. The evolutionary relationship, gene distribution, tandem duplication of *TaANKTM* genes in *Triticeae* species, and expression pattern of these genes in response to different biotic stresses were systematically analyzed. The technology of barley stripe mosaic virus-induced gene silencing (BSMV-VIGS) was used to verify the functions of selected *TaANKTM*-type genes in wheat powdery mildew resistance, and the results showed that silencing of *TaANKTM2A-5* compromised powdery mildew resistance in common wheat Bainongaikang58 (AK58). These findings not only help to understand the evolutionary process of *ANKTM* genes, but also provide gene resources for disease resistance breeding.

## Materials and methods

### Plant materials and *Blumeria graminis* f. sp. *Tritici* race preparation

Common wheat AK58 was used for the BSMV-VIGS and gene expression analysis assays, and was developed and maintained by the Henan Institute of Science and Technology (Xinxiang, China). For the BSMV-VIGS assay, AK58 was grown in a growth cabinet at 70% relative humidity with a 14 h light/10 h dark cycle at 16/12°C. For gene expression analysis, AK58 was grown in a growth cabinet at 70% relative humidity with a 14 h light/10 h dark cycle at 22/18°C. Mixed races of *Blumeria graminis* f. sp. *Tritici* (*Bgt*) were collected from an agricultural field in Xinxiang (China) and maintained on seedlings of the highly susceptibility wheat variety, Sumai 3, and cultured in the light incubator at 70% relative humidity with a 23°C/14 h light and 18°C/10 h dark cycle. The leaves of three individuals were collected at 0, 2, 6, 12, 24, 36, and 48 h post inoculation for RNA extraction.

### Expression analysis of *TaANKTMs* by quantitative real-time reverse transcription-polymerase chain reaction

Total RNAs were extracted from collected samples using the RNA isolation kit, Total RNA Extraction Reagent (Vazyme, Nanjing, China), following the manufacturer’s protocol. The RNAs were reverse-transcribed using HiScript II 1st Strand cDNA Synthesis Kit (Vazyme). The qRT-PCR was performed using AceQ qPCR SYBR Green Master Mix (Vazyme) on a LC 480II platform (Roche, Germany). The procedure used was as follows: 95°C for 5 min, followed by 40 cycles at 95°C for 10 s, 60°C for 20 s. The comparative 2^–ΔΔCT^ method was used to quantify relative gene expression. All the oligonucleotide primers used in this study (Supplementary Table 1) were synthesized by Gene create Corporation (Wuhan, China). The wheat *TaTubulin* gene was used as an internal control.

### BSMV-VIGS

To silence the corresponding *TaANKTM* genes, fragments of the three selected *TaANKTM2A-5*/*TaANKTM3A-2*/ *TaANKTM6A-1* genes with the length of 234 bp, 242 bp, and 276 bp were amplified with corresponding primer pairs (Supplementary Table 1). Each of the target fragment was inserted into the γ-strain of BSMV by the homogenous recombination method to produce BSMV:*TaANKTM* vectors. The second fully expanded leaves of AK58 were infected with the *in vitro* transcribed (mMESSAGEmMACHINE T7, Invitrogen, Waltham, MA, USA) viruses BSMV:*TaANKTM2A-5*, BSMV:*TaANKTM3A-2* and BSMV:*TaANKTM6A-1*, while seedlings infected with BSMV:*TaPDS* and BSMV:*γ* served as controls. The infected plants were grown at 23°C, with a 14 h light/10 h dark cycle environment condition with 70% relative humidity. The fourth fully unfolded leaves with visible viral infection symptoms were detached and placed on 6BA-plate with mixed race of *Bgt* spores to evaluate disease resistance. The inoculated leaves were cultured in a light incubator with a cycle of 14 h light/22°C and 10 h dark at 18°C for 6 days. Target genes silencing efficiency were evaluated by qRT-PCR using the corresponding primer pair *TaANKTM*-Q (Supplementary Table 1).

### Identification of the *ANKTM* genes in Triticeae species

The genomic data for *T. aestivum* (Chinese Spring) was obtained from IWGSC^1^ (IWGSC, 2018). The data for *Triticum urartu* (Tu 2.0) analysis was downloaded from the MBKBase website^2^ (Ling et al., 2018). Data for *Triticum dicoccoides* (WEWSeq_v.1.0), *Hordeum vulgare* (IBSC_v2), *Aegilops tauschii* (Aet_v4.0), and *Arabidopsis thaliana* (TAIR10) were downloaded from the Ensemble Plants website^3^ to construct a local database. The typical ANK domains (PF00023, PF12796, PF13606, PF13637, PF13857) used as the search models were downloaded from the Pfam database^4^ (El-Gebali et al., 2019). A new hidden Markov model (HMM) was built to ensure the search results were reliable. A high-quality protein set (*E*-value <1 × 10^−20^) with intact ANK and TM domains was obtained by the raw ANKTM HMM, and then used to construct a specific ANKTM HMM using hmmbuild from the HMMERv3 suite (Lozano et al., 2015; Xu et al., 2021). The specific ANKTM HMM was used to select the ANKTM protein, and as a result, the proteins with an *E*-value lower than 0.001 were retained. Both the conserved domains^5^ (Lu et al., 2020) and SMART^6^ (Simple Modular Architecture Research Tool; Letunic et al., 2021) were used to recheck the candidate ANKTM protein sequences. When a gene contained multiple transcripts, the longest transcript was retained for further analysis. The analyzed genes were renamed sequentially according to their species and chromosomal distributions on the chromosomes. The gene names and their corresponding gene IDs are listed in Supplementary Table 2.

### Phylogenetic, chromosome localization, gene duplication and micro-collinearity analyses

All the full length ANKTM protein sequences were aligned by ClustalW with the default options in MEGA X and a phylogenetic tree was constructed using the Maximum Likelihood method with 1,000 bootstrap replicates (Kumar et al., 2018). The EvolView^7^ was used to visualize the phylogenetic tree (He et al., 2016). Multiple Collinearity Scan toolkit (MCScanX) was used to identify gene duplication (Wang et al., 2012). The shinyCircos software^8^ was used to express gene duplication events, the syntenic relationship of the gene pairs, and the chromosome localization of the analyzed genes (Yu et al., 2018). TGT (Triticeae-Gene Tribe^9^) was used to trace the evolutionary history of the target genes and for gene pair analyses (Chen et al., 2020b).

### RNA-seq expression analysis

RNA-seq data of 145 wheat *TaANKTM* genes were downloaded from WheatOmics^10^ (Ma et al., 2021). Data for biotic stresses responses (powdery mildew and stripe rust) and after elicitation with PAMPs (chitin and flg22) were collected from N9134 (powdery mildew and stripe rust-resistant wheat) and Chinese Spring, respectively (Zhang et al., 2014a). The relative expressions of each *TaANKTM* gene in response to the different stresses were presented as a heat map constructed with TBtools (Chen et al., 2020a).

## Results

### Genome-wide identification and phylogenetic relationship analysis of the *ANKTM* genes in Triticeae species

**Figure 1:**
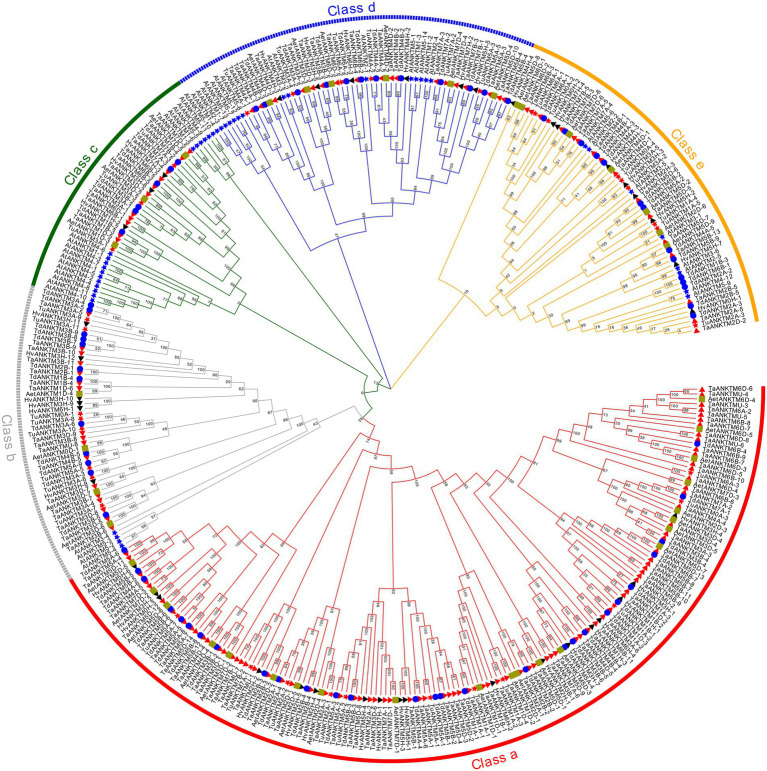
Phylogenetic relationship analysis of 381 ANKTM proteins from *Triticum aestivum*, *Triticum dicoccoides*, *Aegilops tauschii*, *Triticum urartu*, *H. vulgare* and *Arabidopsis.* The phylogenetic tree was built using the Maximum likelihood method with 1,000 bootstrap replicates by MEGA X. The diverse classes of ANKTM proteins were marked with different colors. The ANKTM proteins of *T. aestivum*, *T. dicoccoides*, *A. tauschii*, *T. urartu, H. vulgare*, and *Arabidopsis* were represented by red triangles, blue circles, yellow squares, red stars, black triangles and blue stars, respectively. Gene IDs of all the analyzed genes can be found in Supplementary Table 2.

In the present study, 145, 72, 36, 42, 42, and 44 *ANKTM* genes were identified from *T. aestivum*, *T. dicoccoides, T. urartu*, *Ae. tauschii*, *H. vulgare* and *Arabidopsis*, respectively. To study the evolutionary relationships of the ANKTM proteins, all the above 381 ANKTM protein sequences were used to construct a phylogenetic tree (Figure 1). ANKTM is divided into five classes (class a-e). Among them class a only contains proteins from Triticeae species, while the remaining classes include proteins from Triticeae species and *Arabidopsis*. Within classes b–e, the ANKTM members of Triticeae species are clustered together, in sub-classes distinct from those of *Arabidopsis*, indicating that the *ANKTM* genes of monocots and eudicots had experienced significant differentiation in the evolutionary process. The ANKTM protein sequences of the Triticeae species in all clades of the phylogenetic tree are highly similar, indicating that evolution of the *ANKTM* genes was relatively conservative after Triticeae species speciation.

The numbers of *ANKTM* in each class of all the analyzed species are shown in Table 1. As a heterohexaploid species, wheat resulted from two rounds of hybridization (Shewry, 2009; Zhou et al., 2020). Generally, the number of gene family members in *T. aestivum* (AABBDD) and *T. dicoccoides* (AABB) is about 3 and 2 times that of other diploid Triticeae species, respectively. In this study, the number of *ANKTM* genes in *T. dicoccoides* was 2, 1.71, and 1.71 times that in *T. urartu*, *Ae. tauschii* and *H. vulgare*, respectively, whereas the quantity of *ANKTM* in wheat was 4.03, 3.45, 3.45, and 2.01 times greater that in *T. urartu*, *Ae. tauschii*, *H. vulgare* and *T. dicoccoides*, respectively. This is partly due to the polyploidization of common wheat. However, even after the ploidy level is considered, the number of *ANKTM* genes in common wheat was significantly higher than that in diploid and tetraploid species. On the contrary, the number of *ANKTM* in *T. dicoccoides* was slightly less.

**Table 1:** Numbers of *ANKTMs* in the five analyzed Triticeae species and *Arabidopsis* genomes in total and each class.

Genome	Total number	Subgroup				
		Class a	Class b	Class c	Class d	Class e
*Hordeum vulgare* (HH)	42	18	6	3	6	9
*Triticum urartu* (AA)	36	11	8	3	6	8
*Aegilops tauschii* (DD)	42	22	4	3	6	7
*Triticum dicoccoides* (AABB)	72	28	12	6	12	14
*Triticum aestivum* (AABBDD)	145	84	17	11	17	16
*Arabidopsis*	44	0	4	17	15	8
Total	381	163	51	43	62	62

For the dicotyledonous plant *Arabidopsis*, most of the *AtANKTMs* were mainly distributed in class c and class d, accounting for 72.73% of the total *AtANKTM* genes, while the proportion of Triticeae *ANKTM* genes in these two classes was only 21.66%. Overall, 48.73% of Triticeae *ANKTM* genes were concentrated in class a (Table 1). Class a and class b had significant variation in the proportion of *ANKTM* among the different Triticeae species (Table 1). The numbers of *ANKTM* genes of wheat in class d and class e were below the expected 3:2 or 3:1 ratio compared with the analyzed tetraploid and diploid species, respectively. Conversely, the numbers of wheat *ANKTM* in classes a and class c were significantly larger than expected. The number of *TaANKTM* in class a of *T. aestivum* was about 3, 3.82, 7.64 times that in *T. dicoccoides*, *Ae. tauschii* and *T. urartu*, respectively (Table 1); and in class c the number of *TaANKTM* from *T. aestivum* was about 1.83, 3.66, 3.66 times that in *T. dicoccoides*, *Ae. tauschii* and *T. urartu*, respectively (Table 1); suggesting that wheat *ANKTM* in class a and class c underwent gene expansion.

### Chromosome distribution and gene duplication

The chromosome and subgenome distribution of *ANKTMs* from the five Triticeae species are shown in Table 2. *ANKTM* genes were generally equally distributed among the chromosomes, except the homologous groups 3 and 5, which contained significantly more genes relative to the other homologous groups. The *ANKTM* gene number in the B and D subgenomes were higher than that of the A subgenome (Table 2). From diploid to tetraploid and even hexaploid, the *ANKTMs* number on chromosomes 1A and 3A were decreased whereas the *ANKTMs* number on chromosomes 4A and 7A were increased (Table 2). Compared with *T. urartu*, *Ae. tauschii*, *T. dicoccoides* and *H. vulgare*, which contained 36, 42, 72 and 42 *ANKTMs* genes, respectively, the common wheat family of *ANKTMs* was remarkably large, with 145 members. The data suggest that the evolution of *ANKTM* genes in Triticeae was a complex evolutionary process and that the expansion of *ANKTMs* in common wheat possibly involved more than can be expected from two rounds of polyploidization.

**Table 2:** Numbers of *ANKTMs* from different Triticeae species in each of the chromosomes.

Chromosome	*Triticum aestivum*			*Triticum dicoccoides*		*Triticum urartu*	*Aegilops tauschii*	*Hordeum vulgare*	Total
	A	B	D	A	B	A	D	H	
Chr.1	2	4	6	3	4	5	4	3	31
Chr.2	5	5	2	3	5	3	6	5	34
Chr.3	6	11	9	6	9	11	7	12	71
Chr.4	5	5	5	6	5	2	4	4	36
Chr.5	7	13	14	9	9	5	9	7	73
Chr.6	2	11	8	2	4	3	5	4	39
Chr.7	6	5	6	4	3	3	6	4	37
Total	33	54	50	33	39	32	41	39	321
*Unknown	8					4	1	3	16

*The genes that were assigned to unknown chromosome.

**Figure 2:**
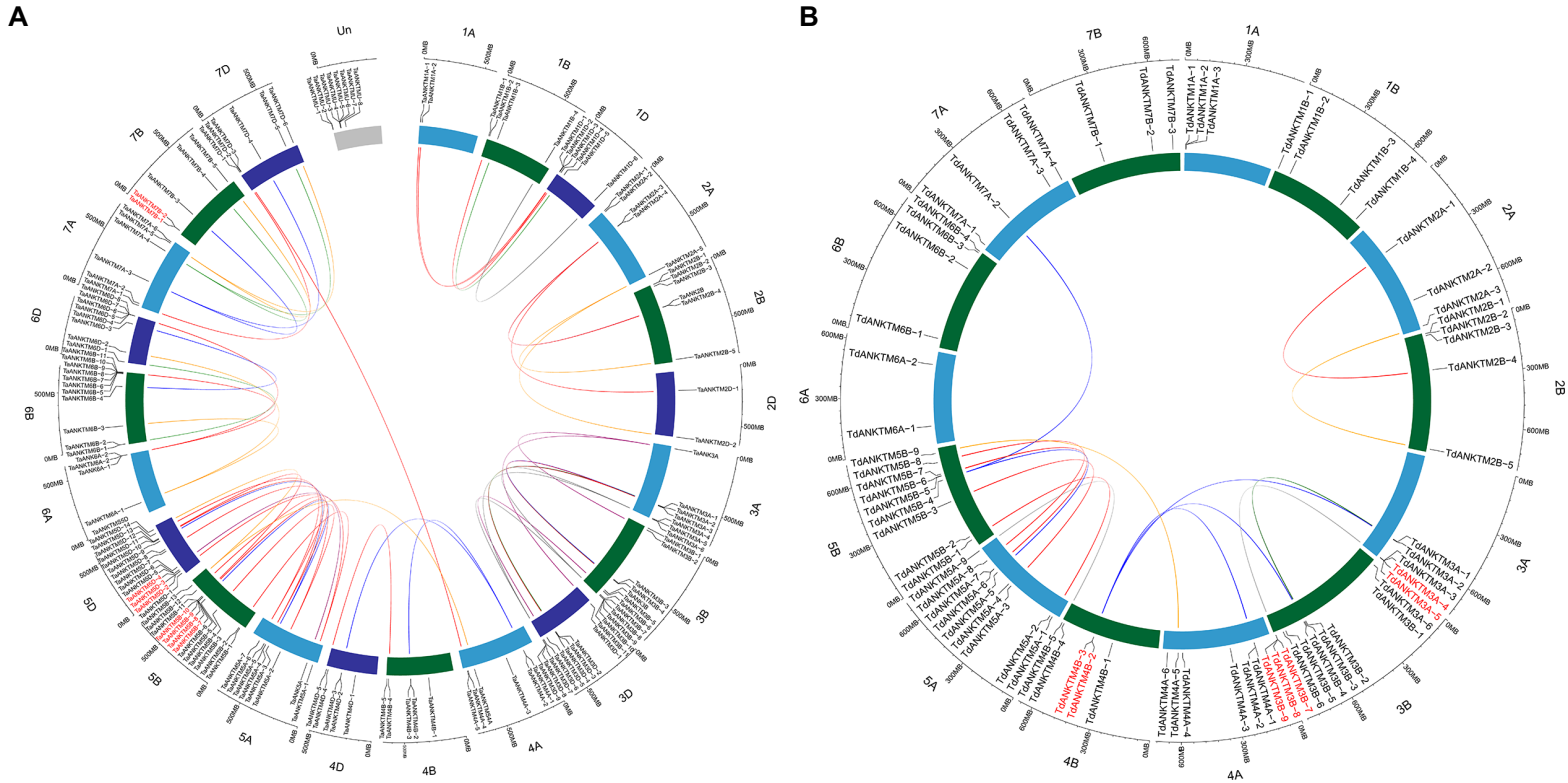
Homologous gene pairs, tandem duplication and location of *ANKTM* genes in wheat and *T. dicoccoides* genomes. All *ANKTM* genes were mapped to their respective loci in the wheat **(A)** and *T. dicoccoides* **(B)** genome in a circular diagram using shinyCircos. Homologous genes were inferred by TGT and linked with specific colors. Tandem duplication was analyzed by MCScanX, and the genes of red font indicated these genes were produced by tandem duplications. Subgenomes A and B are indicated by light blue and dark green, respectively; subgenomes D of wheat is indicated by dark blue.

During the evolution of plant genome and genetic systems, gene duplications have been one of the leading causes for the expansion of gene families (Cannon et al., 2004; Wang et al., 2021; Lin et al., 2022). To further understand the evolution of *ANKTM* genes in wheat and *T. dicoccoides*, the tandem duplications of the common wheat, *T. dicoccoides* and *T. urartu* of the *ANKTM* family were investigated. In this study, the MCScanX analysis was used to investigate the tandem duplication of the *ANKTM* gene family, and shinyCircos software was used to show the tandem duplication genes, the syntenic relationship of the gene pairs and their respective loci in the wheat and *T. dicoccoides* genomes (Figure 2). Tandem duplication analysis of *ANKTM* indicated that wheat, *T. dicoccoides* and *T. urartu* had experienced three, three and two tandem duplication events, respectively (Figure 2; Supplementary Table 2). The above indicates that *ANKTM* is active in the process of evolution and has experienced multiply tandem duplications at different evolutionary stages. The tandem duplication of *ANKTM* in wheat are located on chromosome 5D (*TaANKTM5D-2*, *TaANKTM5D-3* and *TaANKTM5D-4*), 5B (*TaANKTM5B-7*, *TaANKTM5B-8*, *TaANKTM5B-9* and *TaANKTM5B-10*) and 7B (*TaANKTM7B-1* and *TaANKTM7B-2*; Figure 2A). In *T. dicoccoides* are located on chromosome 3A (*TdANKTM3A-4* and *TdANKTM3A-5*), 3B (*TdANKTM3B-7*, *TdANKTM3B-8* and *TdANKTM3B-9*) and 4B (*TdANKTM4B-2* and *TdANKTM4B-3*; Figure 2B). The tandem duplications of *ANKTM* in *T. urartu* are located on chromosome 1A (*TuANKTM1A-4* and *TuANKTM1A-5*) and 3A (*TuANKTM3A-9* and *TuANKTM3A-10*; Supplementary Table 2). The above results may partly explain why the *ANKTM* genes in Triticeae are mainly concentrated in homologous groups 3 and 5, and the number in the B and D subgenomes are higher than that of the A subgenome. Interestingly, the tandem *ANKTM* duplicates in wheat all clustered in class a, whereas those in *T. dicoccoides* all clustered in class b and in *T. urartu* were clustered in classes b and e (Figure 1). This may also be part of the reason that the number ratio of *ANKTM* between wheat and other diploid Triticeae species in class a is higher than 3:1, and in class b and class e are lower than 3:1 (except class b of *Ae. tauschii*). The above indicates that besides the genome polyploidization result in the increase of *ANKTM* gene number, tandem duplication is also a mechanism for the subfamily expansion. A few *ANKTMs* from *T. dicoccoides* and wheat produced gene pairs across homologous groups. For example, *TdANKTM4A-5* of class e was located on the fourth homologous group. However, the gene pairs of *TdANKTM4A-5* from B (*TdANKTM5B-9*) subgenomes of *T. dicoccoides* was located on the fifth homologous group (Figure 2B). This may be due to structural rearrangements of chromosomes 4A–5A–7B in the formation of the *Triticum-Aegilops* joint genus in two major translocation events (Chen et al., 2020b).

### Micro-collinearity analysis of Triticeae *ANKTMs*

**Figure 3:**
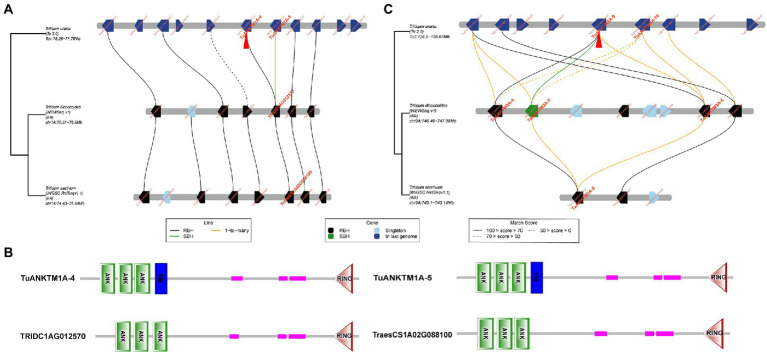
Micro-collinearity analysis by TGT to track the evolutionary history of the tandem duplicated *TuANKTM* homologs. **(A)** The tandem repeats *TuANKTM1A-4* and *TuANKTM1A-5* were used as query genes. The micro-collinearity relationship showed that homologs of the analyzed *TuANKTMs* were found in all investigated genomes, and the homologous of *TuANKTM1A-4* and *TuANKTM1A-5* was *TRIDC1AG012570* in *T. dicoccoides* and *TraesCS1A02G088100* in wheat, respectively. **(B)** Conservative domain prediction of TRIDC1AG012570 and TraesCS1A02G088100 proteins. The conserved domain prediction indicates that TRIDC1AG012570 and TraesCS1A02G088100 are ANK proteins with a RING finger domain. Abbreviations: ANK, ankyrin repeat; TM, transmembrane domain; RING, RING finger domain. **(C)** The tandem repeats *TuANKTM3A-9* and *TuANKTM3A-10* were used as query genes. *TuANKTM3A-9* and *TuANKTM3A-10* from *T. urartu* had a “1-to-many” pairwise homology with *TdANKTM3A-4*, *TdANKTM3A-5* and *TdANKTM3A-6* in the A-subgenome of *T. dicoccoides*; meanwhile the homologous of the above genes in the collinearity regions of common wheat was just *TaANKTM3A-5*. Blackline, 1-to-1-mutual-best. Greenline, 1-to-its-best. Yellowline, 1-to-many. RBH, reciprocal best hits; SBH, single-side best hits.

The tandem duplications of *ANKTM* in *T. urartu* are located on chromosomes 1A and 3A, and the *ANKTMs* on chromosomes 1A and 3A of *T. urartu* are more than that of *T. dicoccoides* and wheat (Table 2; Supplementary Table 2). To trace the evolutionary history of the target genes, a micro-collinearity analysis was performed to help understand their evolution in a local region (Chen et al., 2020b). When the tandem duplication produced genes of *TuANKTM1A-4* and *TuANKTM1A-5* were used as query genes, the results showed that its neighboring genes were relatively conserved across the investigated genomes (Figure 3A), and homologs of *TuANKTM1A-4* and *TuANKTM1A-5* were found in the collinearity regions of the A subgenome of *T. dicoccoides* and wheat was *TRIDC1AG012570* and *TraesCS1A02G088100*, respectively (Figure 3A). The prediction of protein conserved domains indicated TRIDC1AG012570 and TraesCS1A02G088100 as ANK proteins with a RING finger domain, but no TM domain (Figure 3B), belonging to the ANKRF type protein subgroup (Vo et al., 2015). Using the tandem repeats of *TuANKTM3A-9* and *TuANKTM3A-10* as query genes, the micro-collinearity relationship showed that both *TuANKTM3A-9* and *TuANKTM3A-10* from *T. urartu* had a “1-to-many” pairwise homology with *TdANKTM3A-4*, *TdANKTM3A-5* and *TdANKTM3A-6* in the A-subgenome of *T. dicoccoides* (Figure 3C). However, in the collinearity regions of common wheat, only *TaANKTM3A-5* was found to show homology (Figure 3C). Among the subgenomes of the three analyzed species, the number of genes in the micro-collinearity region varies greatly. Nevertheless, the number of collinear genes found in this region of wheat is much less than that of *T. dicoccoides* and *T. urartu* (Figure 3C). The above suggests that *ANKTM* type genes evolved into other types of *ANK* genes in the process of Triticeae evolution and that the evolution of *ANKTM* type genes is complex. However, we also cannot exclude the possibility that some of the diploid and tetraploid species experienced gene loss or generated genes by duplication after *T. aestivum* speciation.

### Expression patterns analysis of *TaANKTMs*

**Figure 4:**
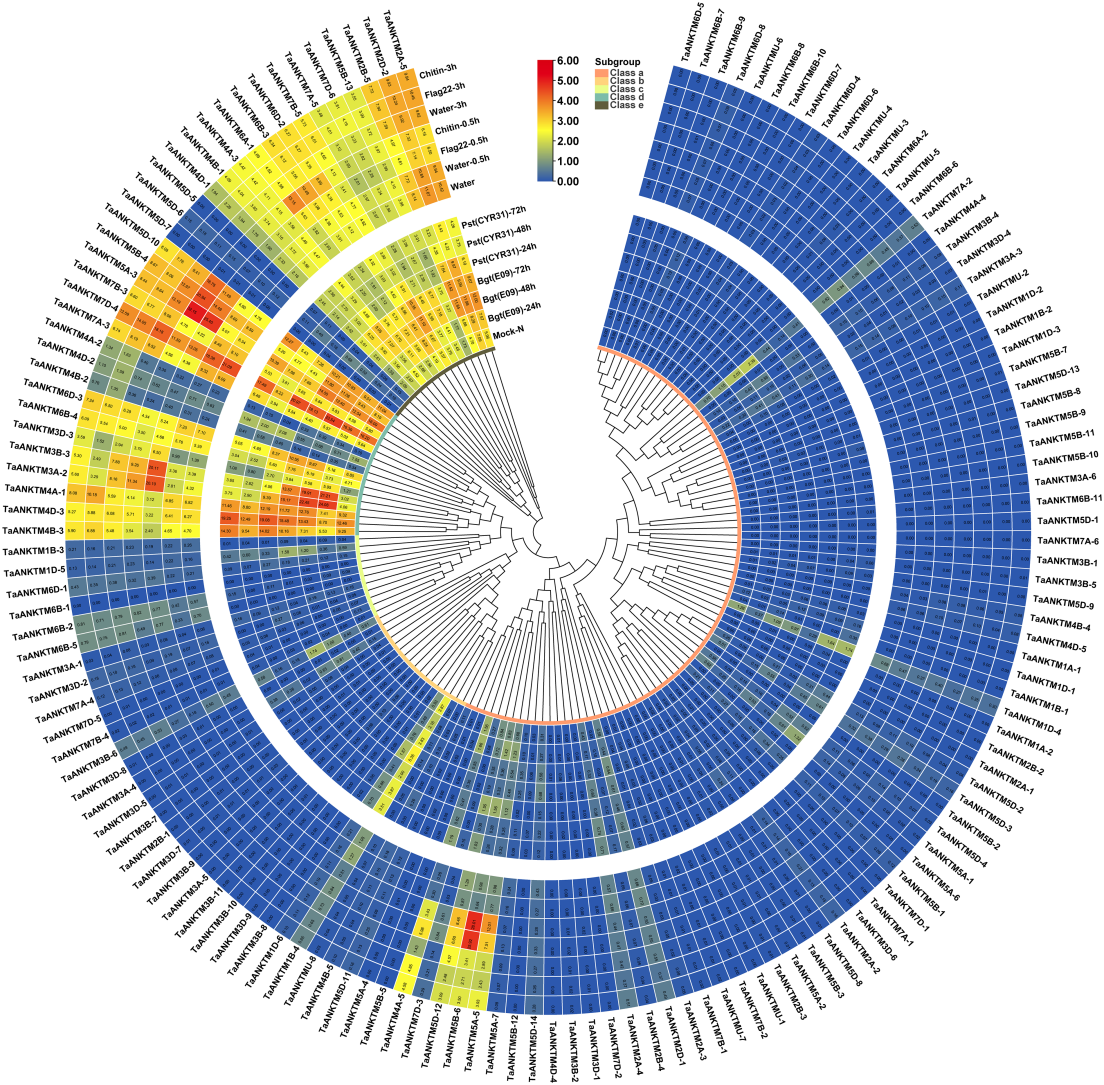
Heat map of the expression profiling of wheat *ANKTM* genes under different stress. The color scale bar represents the expression values (in log2-based tags per million values) of the genes, and the values in square frames represent the tags per million values. The phylogenetic tree was constructed using the neighbor-joining method with 1,000 bootstrap replicates by MEGA X. *Bgt*, *Blumeria graminis* f. sp. *tritici*; *Pst*, *Puccinia striiformis* f. sp. *tritici*; Mock-N, Disease-resistant wheat varieties N9134 without the infection of *Bgt* and *Pst*.

The expression patterns of genes are helpful in predicting their potential biological functions (Hu et al., 2022). To elucidate the potential role of *TaANKTMs* in biotic stress, their expression patterns were studied by *in silico* expression profiling. Expression patterns of *TaANKTMs* under two biotic stresses (powdery mildew pathogen and stripe rust) and two PAMPs (flag 22 and chitin) were analyzed using the wheat RNA-seq data from public databases (Ma et al., 2021). The data are displayed in a heat map (Figure 4). Interestingly, the expression of almost all *TaANKTM* genes in classes a-c could not be detected under the different treatments. In contrast, most of the genes in class d and class e are expressed, and some of them show differential responses to infections by *Bgt*, *Puccinia striiformis* f. sp. *tritici* (*Pst*) or elicitation with chitin or flg22 (Figure 4). For example, the gene pairs of *TaANKTM3A-2* (*TaANKTM3B-3* and *TaANKTM3D-3*) and *TaANKTM6A-1*(*TaANKTM6B-3* and *TaANKTM6D-2*) were up-regulated upon *Bgt* or *Pst* infection at 24 h and were also up-regulated upon flg22 and chitin treatment at 0.5 h. The gene pairs, *TaANKTM7B-5* (*TaANKTM7A-5* and *TaANKTM7D-6*), were up-regulated after *Bgt* infection, down-regulated after the infection with *Pst*, but displayed only insignificant alterations in expression after chitin and flg22 treatments. The expression of the gene pair, *TaANKTM5A-3* (*TaANKTM5B-4* and *TaANKTM5D-10*), was obviously up-regulated upon the treatments of chitin and flg22. The gene pair of *TaANKTM7A-3* (*TaANKTM7B-3* and *TaANKTM7D-4*), was obviously down-regulated upon flg22 and chitin treatment at 0.5 h; specially, the expression of *TaANKTM7D-4* was up-regulated upon *Bgt* infection at 48 h and *Pst* infection at 24 h. The expression of *TaANKTM6B-4* and *TaANKTM6D-3* were slightly up-regulated after *Bgt* infection at 48 h and *Pst* infection at 24 h, however, were down-regulated upon flg22 and chitin treatment at 0.5 h. The gene pair of *TaANKTM4A-1*(*TaANKTM4B-3* and *TaANKTM4D-3*) was slightly down-regulated and up-regulated upon *Bgt* and *Pst* infection at 24 h, respectively; and was down-regulated upon flg22 and chitin treatment at 0.5 h. In addition, the expression levels of the gene pairs *TaANKTM2A-5* (*TaANKTM2D-2* and *TaANKTM2B-5*) were up-regulated after infection by *Bgt* and *Pst* and transiently down-regulated after treatments with chitin and flg22 at 0.5 h and returned to the pretreatment levels at 3 h. The RNA-Seq data suggests the above genes can response to rust, powdery mildew infections or PAMP elicitors; however, the absolute times of relative expression change of some genes after *Bgt* infection were small, and change multiples were less than two (Figure 4). Therefore, the genes *TaANKTM2A-5* (*TaANKTM2D-2* and *TaANKTM2B-5*) and *TaANKTM6A-1* (*TaANKTM6B-3* and *TaANKTM6D-2*; class e), and *TaANKTM3A-2* (*TaANKTM3B-3* and *TaANKTM3D-3*; class d) induced by *Bgt* obviously from the RNA-Seq data were selected for further expression analysis by qRT-PCR following powdery mildew stress.

**Figure 5:**
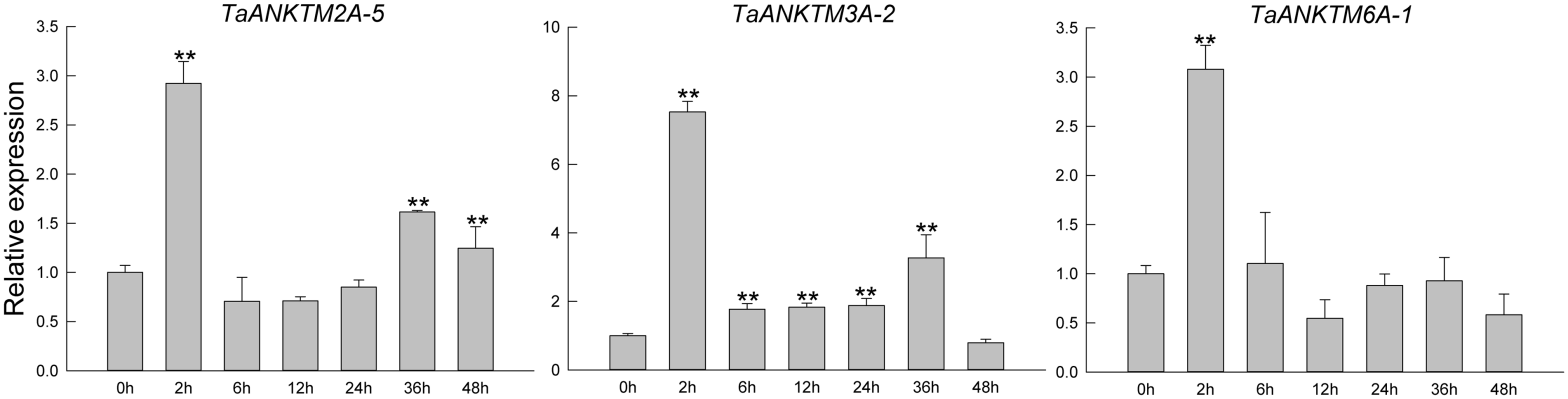
Expression profiling of three *TaANKTMs* in common wheat AK58 after *Bgt* inoculation by qRT-PCR. Relative expression of three *TaANKTMs* in response to *Bgt*. Data were normalized to the *TaTubulin* gene. The values are the means of three technical replicates of one biological experiment. Asterisks indicate significant differences (assessed using Duncan’s honestly significant difference test), **p < 0.01. All the raw data for qRT-PCR are listed in Supplementary Table 3.

Because of the high sequence similarity of the three copy genes in different subgenomes of wheat, qRT-PCR primers could not effectively distinguish the three copy genes; therefore, the gene of subgenomes A was used to represent the relative expression of the three copy genes. After *Bgt* inoculation, the expression patterns of *TaANKTM2A-5*, *TaANKTM3A-2*, and *TaANKTM6A-1* were similar, the relative expression levels of the three genes were rapidly up-regulated and reached to the expression peak at 2 h (Figure 5). The relative expression levels of *TaANKTM2A-5* returned to the original level at 6, 12 and 24 h, and significantly up-regulated at 36 h and 48 h (Figure 5). For *TaANKTM3A-2*, the relative expression reached a new peak at 36 h and then returned to the original expression level at 48 h (Figure 5). For *TaANKTM6A-1*, the relative expression reached to the expression peak at 2 h and then returned to the original level at 6 h, and there was no significant change at each subsequent time point (Figure 5). Since all the three genes can respond to the induction of *Bgt*, they were therefore selected for further analysis of their potential biological functions.

### Silencing of *TaANKTM2A-5* compromises powdery mildew resistance in common wheat AK58

**Figure 6:**
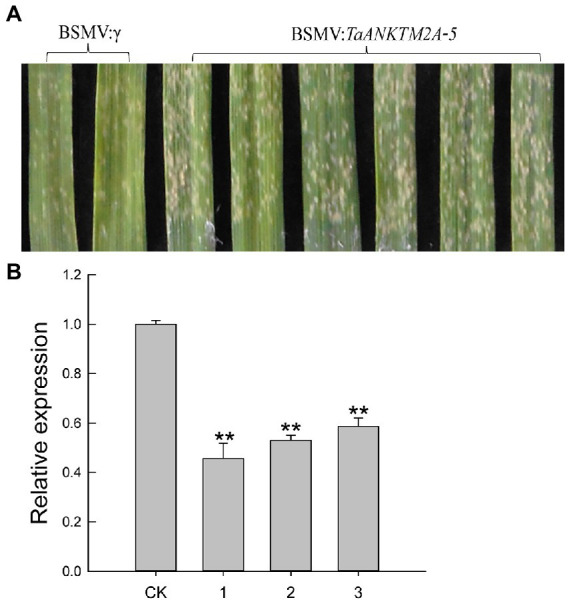
Functional analysis of *TaANKTM2A-5* by Barley stripe mosaic virus-based virus-induced gene silencing (BSMV-VIGS) in AK58. **(A)** BSMV: *TaANK2A-5* infected plants were inoculated with *Blumeria graminis* f. sp. *Tritici*, and their leaves were photographed 6 days post-inoculation. BSMV:γ infected plants were performed as controls. The experiment was repeated independently three times, and the same results were obtained. **(B)** Expression of *TaANKTM2A-5* in BSMV: *TaANKTM2A-5*-infected leaves was compared with that in BSMV:γ-infected controls of AK58. CK represents plants inoculated with BSMV:γ, and 1–3 represents plants inoculated with BSMV: *TaANK2A-5*. Significant differences assessed using Duncan’s honestly significant difference test, **p < 0.01.

In order to further explore the potential roles of *TaANKTM2A-5*, *TaANKTM3A-2*, and *TaANKTM6A-1* in the resistance to powdery mildew disease in the common wheat variety, AK58, constructs for their virus (BSMV) induced silencing were produced. Six days after *Bgt* infection, *TaANKTM2A-5*-silenced leaves were seen to be more susceptible to *Bgt* than those of the control (BSMV:γ-innoculated plants; Figure 6A). *TaANKTM3A-2*- and *TaANKTM6A-1*-silenced leaves showed no obvious difference to *Bgt* infection than those from BSMV:γ-innoculated individuals (Supplementary Figures 1A1,A2). The expression levels of *TaANKTM2A-5*, *TaANKTM3A-2* and *TaANKTM6A-1* were assessed by qRT-PCR and were shown to be significantly decreased by 2–5-fold (Figure 6B; Supplementary Figures 1B1,B2). Therefore, silencing the *TaANKTM2A-5* gene could compromise the resistance of AK58 to powdery mildew.

## Discussion

### Tandem duplication is one of the major mechanisms for *ANKTM* gene subfamily expansion

In the process of evolution, duplicated genes can experience functional divergence, which is essential for speciation and environmental adaptability (Prince and Pickett, 2002; Hittinger and Carroll, 2007). As a heterologous hexaploid species, wheat resulted from two rounds of hybridizations and has experienced complex evolutionary mechanisms (Shewry, 2009; Zhou et al., 2020), which makes it more challenging to explore its evolutionary relationships and functional genomics. The recent rapid development of interdisciplinary bioinformatics and high-quality genome assembly of *Triticeae* species (Avni et al., 2017; Luo et al., 2017; IWGSC, 2018; Ling et al., 2018) have provided the opportunity for a more detailed study of the phylogenetics of *ANKTM* genes and inferring how gene members of this subfamily replicated and expanded.

In the present study, bioinformatics methods were used to analyze the *ANKTM* gene family in Triticeae species and investigate the expansion mode of *ANKTM*. The number of *ANKTM* genes identified from the whole genome of wheat (BBAADD), *T. dicoccoides* (BBAA), *T. urartu* (AA), *Ae. tauschii* and *H.vulgare* (HH) were 145, 72, 36, 42 and 42, respectively (Table 1). The increase in *ANKTM* numbers in wheat was proportionally larger than what could be expected from increase in its ploidy. Furthermore, the numbers of *ANKTM* in class a and class c of wheat were significantly larger than expected (Figure 1; Table 1). Several tandem duplication events occurred in wheat, *T. dicoccoides* and *T. urartu ANKTM* genes (Figure 2; Supplementary Table 2), and most of the tandem duplication produced genes distributed in the distal chromosome segments. This finding is consistent with that many fast-evolving genes are distributed in these segments and possibly to facilitate the adaption to different conditions (Glover et al., 2015; Chen et al., 2018; Schilling et al., 2020). The tandem duplication produced *ANKTM* genes of wheat all clustered in class a, thus contributing to the unexpectedly high number of wheat *ANKTMs* in class a. Previous studies indicate that the seven *ANKTM*-type genes *At4g03440, At4g03450, At4g03460, At4g03470, At4g03480, At4g03490* and *At4g03500* on chromosome 4, are tandem duplicated genes, which are linked and clustered tightly (Du et al., 2007). The seven genes in this study are *AtANKTM4-1*, *AtANKTM4-2*, *AtANKTM4-3*, *AtANKTM4-4*, *AtANKTM4-5*, *AtANKTM4-6* and *AtANKTM4-7*, respectively (Supplementary Table 2), this consistent with the previous research. The ANKTM protein Lr14a confers leaf rust disease resistance in wheat, and its gene resides in a locus with tandem repeats (Kolodziej et al., 2021). *TaANKTM5B-9* (*TraesCS5B02G352300*) is a homolog of *Lr14a* (Kolodziej et al., 2021), and in this study, we find that *TaANKTM5B-7*, *TaANKTM5B-8*, *TaANKTM5B-9* and *TaANKTM5B-10* are tandem repeats (Figure 2). These results therefore agree with previous studies indicating that the increase of *ANKTM* gene family members in wheat is not only due to the increased ploidy level, but that self-replication is also a major mechanism driving the gene family expansion (Cannon et al., 2004; Du et al., 2007; Kolodziej et al., 2021).

### *TaANKTM2A-5* positively regulates powdery mildew resistance in wheat

Wheat powdery mildew, caused by *Bgt*, is one of the most destructive diseases of wheat (Hu et al., 2018). Cultivating broad-spectrum and durable disease-resistant varieties is an effective and environment-friendly strategy to improve plant diseases (Li et al., 2020). Resistance genes play important roles in disease resistance breeding and are frequently pathogen-specific (Dangl et al., 2013; Saintenac et al., 2018; Xing et al., 2018). However, the natural evolution of new races of the pathogen can overcome the resistance (Dodds and Rathjen, 2010), so that new types of resistance-related genes need to be explored continuously.

Several *ANKTMs* have been identified to be involved in plant immune responses. For example, the *Arabidopsis ANKTM* family gene, *BDA1*, is an important regulator of plant immunity acting downstream of the receptor-like protein *SNC2* and upstream of *NPR1* and *WRKY70* (Yang et al., 2012). The widely studied *Arabidopsis ANKTM, ACD6*, which plays a key role in growth and pathogen defense (Todesco et al., 2010), can form large complexes with the membrane bound PAMP receptors, BAK1 and CERK1 (Tateda et al., 2014; Zhang et al., 2014b). Lr14a encodes an ANKTM-like type protein that confers race-specific leaf rust resistance in wheat (Kolodziej et al., 2021). The NLR protein, YrU1, confers stripe rust resistance in wheat also contains an ANK domain, and the ANK domain of YrU1 may be derived from ANKTM proteins (Wang et al., 2020; Kolodziej et al., 2021). The above studies indicated that some of the ANKTM-type genes are involved in basal resistance and effector-triggered immunity. In this study, the expression of some *TaANKTMs* were found to be differentially responsive to infection by *Pst* or *Bgt*, or challenge by PAMPs (chitin, flg22; Figure 4), which is agreement with the earlier studies. In particular, the expression levels of *TaANKTM* genes *TaANKTM2A-5, TaANKTM3A-2* and *TaANKTM6A-1* displayed interesting responses to infection by *Bgt* and *Pst*, and elicitation by chitin and flg22 (Figure 4), and the relative expression of the three genes were further analyzed by qRT-PCR. The results showed that the expression of *TaANKTM2A-5*, *TaANKTM3A-2*, and *TaANKTM6A-1* in powdery mildew susceptible cultivar AK58 were rapidly up-regulated and reached to the expression peak at 2 h after *Bgt* inoculation (Figure 5). The silencing of the *TaANKTM* genes *TaANKTM3A-2* and *TaANKTM6A-1* produced inconsistent phenotypes and no conclusions could be drawn concerning their effects on the resistance of AK58 to *Bgt* (Supplementary Figure 1). The silencing of *TaANKTM2A-5* did however produce a consistent phenotype and plants were clearly compromised in their resistance to *Bgt* (Figure 6).

Based on the above research, we speculate that the ANKTM type protein may represent a new family of disease resistance related genes in plants. In this study, the function of three *ANKTM* genes on powdery mildew resistance was verified through the BSMV-VIGS assay. In future studies, the function of the *TaANKTM2A-5* gene on wheat powdery mildew needs to be further verified in stable genetically transformed plants, and the three-dimensional structural characteristics of proteins with similar sequences and expression patterns but display different functions will be further analyzed. Findings in this study not only help in understand their evolutionary process, but also provide gene resources for disease resistance breeding.

## Conclusion

In summary, a total of 381 *ANKTM* genes were identified from five Triticeae species and *Arabidopsis*, which could be divided into five classes. Among them class a only contains proteins from Triticeae species, and the numbers of *ANKTM* in class a of wheat are significantly larger than expected even after consideration of the ploidy level. *T. urartu*, *T. dicoccoides* and wheat all experienced tandem duplication events in the evolution process. Furthermore, the tandem duplication produced *ANKTM* genes of wheat all clustered in class a. Tandem duplication is one of the major mechanisms for *ANKTM* gene subfamily expansion. The expression pattern showed that almost all *TaANKTM* genes in classes a-c could not be detected under different treatments. In contrast, most of the genes in class d and class e are expressed, and some of them are responsive to biotic stress. Furthermore, silencing *TaANKTM2A-5* belonging to class e compromised powdery mildew resistance in common wheat AK58. The findings in this study not only help our understanding of the evolutionary process of *ANKTM* genes, but also provide a valuable reference for mining disease resistance genes in the *ANKTM* gene family.

## Data availability statement

The datasets presented in this study can be found in online repositories. The names of the repository/repositories and accession number(s) can be found in the article/Supplementary Material.

## Author contributions

PH and JX designed the experiments and wrote the manuscript. PH, YR, JX, QW, PS, YG, YZ, and HG contributed to the experiments and performed the data analysis. HH and CL revised the manuscript. All authors contributed to the article and approved the submitted version.

## Funding

This work was supported by the National Natural Science Foundation of China (nos. 31901538, 31872129, and 31900240) and the Key Scientific and Technological Research Projects in Henan Province (no. 212102110052).

## Conflict of interest

The authors declare that the research was conducted in the absence of any commercial or financial relationships that could be construed as a potential conflict of interest.

## Publisher’s note

All claims expressed in this article are solely those of the authors and do not necessarily represent those of their affiliated organizations, or those of the publisher, the editors and the reviewers. Any product that may be evaluated in this article, or claim that may be made by its manufacturer, is not guaranteed or endorsed by the publisher.

## Supplementary material

The Supplementary Material for this article can be found online at: https://www.frontiersin.org/articles/10.3389/fpls.2022.943217/full#supplementary-material

## Footnotes

^1^ http://www.wheatgenome.org/

^2^ http://www.mbkbase.org/Tu/

^3^ http://plants.ensembl.org/index.html

^4^ http://pfam.xfam.org/

^5^ https://www.ncbi.nlm.nih.gov/Structure/cdd/wrpsb.cgi

^6^ http://smart.embl-heidelberg.de/

^7^ https://www.evolgenius.info/

^8^ http://shinycircos.ncpgr.cn

^9^ http://wheat.cau.edu.cn/TGT/

^10^ http://wheatomics.sdau.edu.cn/expression/wheat.html
