# Supplementary material for: Identification of ankyrin-transmembrane-type subfamily genes in Triticeae species reveals TaANKTM2A-5 regulates powdery mildew resistance in wheat
Source: Front Plant Sci. 2022 Jul 22;13:943217. doi: 10.3389/fpls.2022.943217 (PMC9353636; doi:10.3389/fpls.2022.943217)
Supplement: Supplementary file 1 [file Table_1.DOCX]

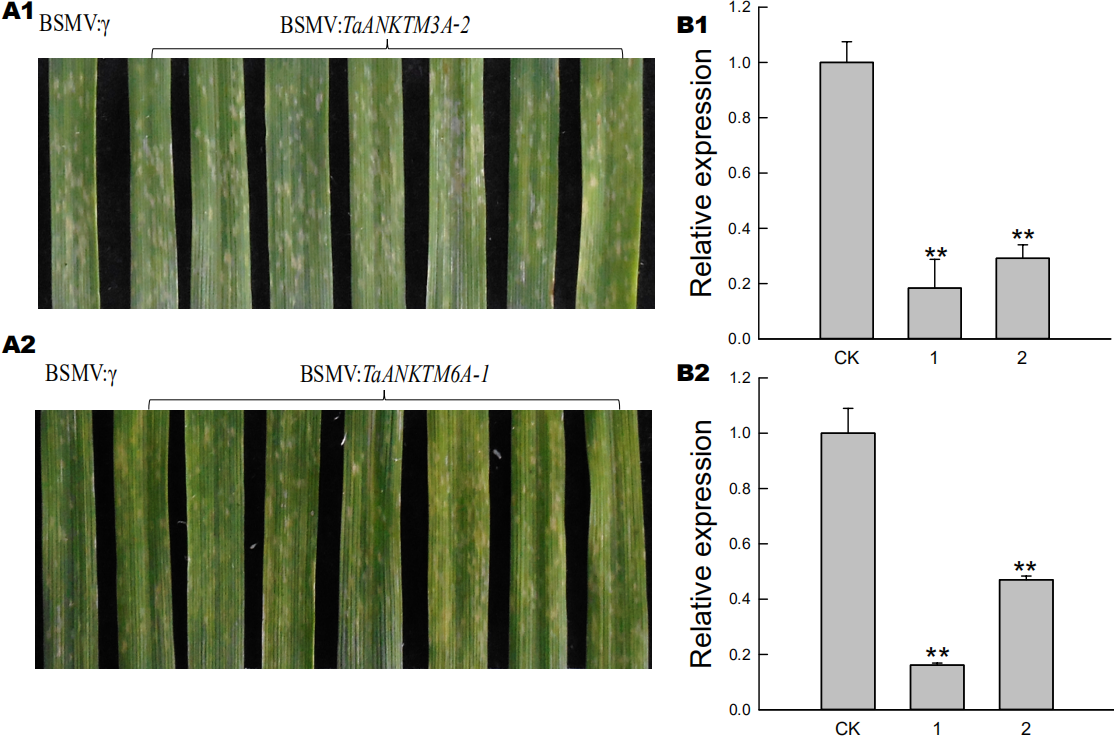


**Supplementary Figure 1. Functional analysis of *TaANKTM3A-2* and *TaANKTM6A-1*** **by Barley stripe mosaic virus-induced gene silencing (BSMV-VIGS) in AK58.**

**(A)** BSMV: *TaANKTM3A-2* **(A1)** and *TaANKTM6A-1* **(A2)** infected individual plants were inoculated with *Blumeria graminis f. sp. Tritici*, respectively, and BSMV:γ were performed as a control. Photographs were taken six days post-inoculation (dpi). **(B)** Expression of *TaANKTM3A-2* **(B1)** and *TaANKTM6A-1* **(B2)** in BSMV:*TaANKTM3A-2*- and BSMV:*TaANKTM6A-1*-infected leaves were compared with that in BSMV:γ-infected controls of AK58, respectively. CK represents plants inoculated with BSMV:γ, and 1-2 represents plants inoculated with BSMV: *TaANKTM3A-2* or BSMV: *TaANKTM6A-1*, respectively. Significant differences assessed using Duncan’s honestly significant difference test, ** P < 0.01.
