# Supplementary material for: Identification of ankyrin-transmembrane-type subfamily genes in Triticeae species reveals TaANKTM2A-5 regulates powdery mildew resistance in wheat
Source: Front Plant Sci. 2022 Jul 22;13:943217. doi: 10.3389/fpls.2022.943217 (PMC9353636; doi:10.3389/fpls.2022.943217)
Supplement: Supplementary file 2 [file Table_2.DOCX]

**Supplementary Table 1. Primer list.**

| Name | Sequence (5’-3’) | Used for |
| --- | --- | --- |
| *TaANKTM2A-5*-VIGS-F | TAGCTGAGCGGCCGCCCCGGGCTTCGAACCTGATGCAGACA | Construct BSMV: *TaANKTM2A-5* vector |
| *TaANKTM2A-5*-VIGS-R | TAGCTGATTAATTAACCCGGGCGCCTTTATGGCCTCTACAA |  |
| *TaANKTM3A-2*-VIGS-F | TAGCTGAGCGGCCGCCCCGGGCATATGCAGTGCCGTTTGAC | Construct BSMV: *TaANKTM3A-2* vector |
| *TaANKTM3A-2*-VIGS-R | TAGCTGATTAATTAACCCGGGCATTTGGCCCTGCAAATACT |  |
| *TaANKTM6A-1*-VIGS-F | TAGCTGAGCGGCCGCCCCGGGAATTGCCATTTCCTCGTCAG | Construct BSMV: *TaANKTM6A-1* vector |
| *TaANKTM6A-1*-VIGS-R | TAGCTGATTAATTAACCCGGGCCTTGGGTGGTGTCTTTTGT |  |
| *TaANKTM2A-5*-QF | CAAGGGAAGGTGCAATTGAT | qRT-PCR and gene silencing efficiency analysis |
| *TaANKTM2A-5*-QR | CTGAGCATTCACGTCTGCAT |  |
| *TaANKTM3A-2*-QF | TGTGCTTTTTGCTACCGTTG |  |
| *TaANKTM3A-2*-QR | GGACTTTGTTTCACCCCTGA |  |
| *TaANKTM6A-1*-QF | ATGTGATCGCTGTGTTGAGC |  |
| *TaANKTM6A-1*-QR | TAAGCCATCCACCAAACGAT |  |
| Ta-Tubulin-F | ATCTCCAACTCCACCAGTGTCG |  |
| Ta-Tubulin-R | TCATCGCCCTCATCACCGTC |  |
